# Machine learning approach for predicting electrical features of Schottky structures with graphene and ZnTiO_3_ nanostructures doped in PVP interfacial layer

**DOI:** 10.1038/s41598-023-41000-z

**Published:** 2023-08-22

**Authors:** Ali Barkhordari, Hamid Reza Mashayekhi, Pari Amiri, Süleyman Özçelik, Şemsettin Altındal, Yashar Azizian-Kalandaragh

**Affiliations:** 1 Faculty of Physics, Shahid Bahonar University of Kerman, Kerman, Iran; 2 Department of Engineering Sciences, University of Mohaghegh Ardabili, Namin, Iran; 3 Department of Engineering Sciences, Faculty of Advanced Technologies, Sabalan University of Advanced Technologies (SUAT), Namin, Iran; 4 Photonics Application and Research Center, Gazi University, 06500 Ankara, Turkey; 5 Department of Photonics, Faculty of Applied Sciences, Gazi University, 06500 Ankara, Turkey; 6 Department of Physics, Faculty of Sciences, Gazi University, Ankara, Turkey

**Keywords:** Materials for devices, Electronics, photonics and device physics, Mathematics and computing, Nanoscience and technology

## Abstract

In this research, for some different Schottky type structures with and without a nanocomposite interfacial layer, the current–voltage (I–V) characteristics have been investigated by using different Machine Learning (ML) algorithms to predict and analyze the structures’ principal electric parameters such as leakage current (I_0_), barrier height (${\varphi }_{B0}$), ideality factor (n), series resistance (R_s_), shunt resistance (R_sh_), rectifying ratio (RR), and interface states density (N_ss_). The interfacial nanocomposite layer is made by composing polyvinyl-pyrrolidone (PVP), zinc titanate (ZnTiO_3_), and graphene (Gr) nanostructures. The Gaussian Process Regression (GPR), Kernel Ridge Regression (KRR), Support Vector Regression (SVR), and Artificial Neural Network (ANN) are used as ML algorithms. The ML techniques training data are obtained using the thermionic emission method. Finally, by comparing the experimental and predicted results, the performance of the different ML algorithms in predicting the electrical parameters of Schottky diodes (SDs) has been compared to find the optimized ML algorithm. The ML predictions of basic electrical parameters by almost all algorithms are in good agreement with the actual values, while the SVR model has predicted closer values to the corresponding actual ones. The obtained results show that the quantity of the leakage current and N_ss_ for MS type SD decreases, and φ_B0_ increases with the interfacial layer usage, especially with graphene dopant.

## Introduction

Depending on the potential barrier height (BH) value, the metal/semiconductor (MS) structure or Schottky diode (SD) with/without an interfacial polymer layer has been known as a rectifying or non-rectifying device^1^. The contact is either Schottky or ohmic when the potential BH value is enough high or low, respectively^2–5^*.* According to Schottky and Mott theory, the back metal ohmic, front metal rectifier contacts, and the work functions of the semiconductor also determine the ohmic and rectifying behavior of these devices^1,4,5^. In p-type semiconductor, to get a rectifier, the work-function value of metal (Φ_m_) value must be less than work-function of semiconductor (Φ_s_), but to get an ohmic contact, Φ_m_ > Φ_s_. In n-type semiconductor the situation was reversed the opposite is the case for an n-type semiconductor. These structures play an important role in all semiconductor devices such as SD with/without an interfacial polymer layer, capacitor, transistor, and solar cells ^3^. The essential scientific and technological difficulty in the MS structures is that their growing performance and cost-effective fabrication is depended on wielding the various suitable interfacial polymer/organic layers with metal/metal-oxide dopants and large-dielectric materials^3–7^. In other words, the main technological problem today is to both reduce the cost and increase the performance. Because polymers are usually cheap, flexible, easy to grow, have high mechanical and dynamic strength, but have low conductivity and dielectric values. However, this problem can be overcome by doping them with a low proportion of metal, metal oxide, and graphene. Therefore, it is worth to noting that, the prepared conventional insulator layers with the old-fashioned techniques inserted between metal and semiconductor layers have not been able to passivate the dangling bonds which activate on the semiconductor surface and reduce the leakage current^6–11^. In addition, sufficient flexibility, high surface space compared to volume rate, large capacity in charge/energy-storage, easy procedure techniques, weightlessness, and suitable stability of dielectric and mechanical characteristics are some of the reasons for improving the electric, dielectric, and optical features of these MS devices with an interfacial polymer/organic layer with/without metal, graphene, and metal-oxide dopants^12–15^. Therefore, controlling and manipulating potential BH is necessary to enhance the performance of the MS structure.

The electrical response of SDs varies depending on the operating environment, which affects its applicability in electronics technology. Moreover, the characterization of electric features of SDs is a long-time and high-cost process due to the sensitive laboratory requirement. So, engineering tools with high reliability are essential, enabling to predict the electrical properties of SDs. On the other hand, there are sometimes significant deviations from the ideal state between the electrical parameters obtained using thermionic emission theory as experimental and the theoretical methods. These deviations are generally due to the inhomogeneity of the barrier and the interfacial layer formed between the M/S interface, the series resistance (R_s_) of the diode, and the interface states/traps (N_ss_) created between the interfacial layer and the semiconductor. Therefore, it is obvious to turn to alternative methods that can reduce part of the volume of experimental tests, leading to cost reduction and time-saving. Currently, the most prominent method to achieve this goal is the use of Machine Learning (ML), and scientific research has not only proven the applicability of ML in most branches of science and technology but also shows us the utility of this powerful scientific tool in the field of Schottky structures and predicting some of their electrical properties. ML is one of the subfields of artificial intelligence that is able to evaluate data samples and make some rules and patterns to extend a simulation model for predicting new data. It is a powerful predictive tool with high accuracy and no need for human decision and intermediation which is applied in many fields. ML consists of extensive types of modeling algorithms to learn the rules and then predict the new data^13^. Recently, the electrical parameters of SDs have been analyzed with the Machine Learning (ML) method in the literature.

Torun et al.^14^ analyzed the I–V characteristics of Au/Ni/n-GaN SD in the temperature range of 40–400 K with 4 algorithms of ML technique. They used Adaptive Neuro Fuzzy System (ANFS), Artificial Neural Network (ANN), Support Vector Regression (SVR), and Gaussian Process Regression (GPR) algorithms for modeling the experimental data of 5192 samples. After obtaining the model error and they compared their performance with each other, and they uncovered that the AFNS model has shown the best performance in both the train and test phases among the utilized models. Ali et al.^15^ modeled the electrical current of heterojunction SDs as a function of voltage and temperature by using an ANN based on the experimental data. They presented that the I–V results predicted by the ANN model are in good agreement with the experimental ones with a suitable accuracy. Güzel et al.^16^ reported the ANN system prediction of electric current of 6H-SiC/MEH-PPV SDs with an interfacial polymer layer in terms of the voltage and temperature. The experimental results were measured at the voltage and temperature ranges of − 3V to + 3V and 100–250 K, respectively. The results predicted by ANN system are incompatible with the experimental ones with a good accuracy and an average deviation of 0.15%. Çolak et al.^17^ used an ANN model with 15 neurons in 1 hidden layer to predict the electric current of a SD depending on the voltage of − 2 V to + 3 V and the temperature of 100–300 K. It was found that the predicted data are in good agreement with experimental results of SD. However, the investigation of ML techniques to determine the electrical parameters of SDs has been limited so far, and more research and investigations are needed, especially to identify and improve appropriate ML algorithms for Schottky structures. Therefore, it should be researched which algorithms have better predictive performance and under the influence of various factors on the characteristics of the input data, they still maintain this advantage, and in addition, how to increase their efficiency by improving and upgrading common algorithms.

In most studies conducted in the Schottky structures that have employed machine learning, the ANN algorithm has been utilized for training and prediction purposes. The obtained results demonstrate that the predicted data closely approximate the actual data with a negligible margin of error^14–17^. In this research, in addition to utilizing the ANN algorithm, we have employed three other algorithms, namely Gaussian Process Regression (GPR), Kernel Ridge Regression (KRR), and Support Vector Regression (SVR), for training and predicting the I–V characteristics of Schottky diodes. The purpose of employing these four algorithms is to examine their accuracy, prediction error, and overall performance by comparing the results of their predictions with each other and with the experimental data. Subsequently, if possible, we aim to identify an algorithm that exhibits the most negligible prediction error compared to the other algorithms employed in this study, thus demonstrating superior performance in most cases.

A perovskite-type ZnTiO_3_ and graphene could be considered as an interfacial layer grown between metal and semiconductor to get MIS type SD, capacitor, and solar cell aplications^18–20^. Therefore, in this study, zinc-titanate and graphene nanostructures have been doped into the PVP as interfacial layer between Al and p-Si layers to enhance the electrical performance of the MS-type SD. Five SDs with the structures of Al/p-Si (MS), Al/PVP/p-Si (MPS1), Al/PVP:Gr/p-Si (MPS2), Al/PVP:ZnTiO_3_/p-Si (MPS3), and Al/PVP:Gr-ZnTiO_3_/p-Si (MPS4) have been manufactured to study the effect of these interfacial polymer layers with/without dopants on the basic electric parameters of the MS type SD. The procedures of the material preparation and fabrication of the SDs have been concisely expressed. Next, the I–V characteristics of the SDs have been measured by the TE method to calculate and analyze the principal electric parameters of the SDs, such as n, BH, I_0_, R_sh_, and R_s_. After that using above mentioned ML algorithms, the I–V characteristics have been predicted, and then above and some other electrical parameters of SDs calculated. Comparing the experimental and predicted results, the performance of the different ML algorithms for accurately predicting the electrical parameters of SD have been evaluated to find the optimized ML algorithm.

## Experimental details

### Materials

The precursors of TiCl_4_, Zn(CH_3_COO)_2_.2H_2_O, and NaOH with a purity of more than 99% were provided by ROYALEX and Merck Companies, respectively. In order to rinse the Si wafer, the compounds of H_2_O_2_, HF, CH_3_COCH_3_, CH_3_OH, and C_2_H_5_OH have been used.

Moreover, the prepared nanocomposites were irradiated by a microwave device at a power range from 100 to 800 W and a fixed frequency of 2450 MHz produced by Samsung: model ME2040/ME201 (Korean company). To measure the I–V characteristics of the SDs have been carried out by KEITHLEY (Model 2450).

### Synthesis of ZnTiO_3_ nanostructures

**Figure 1:**
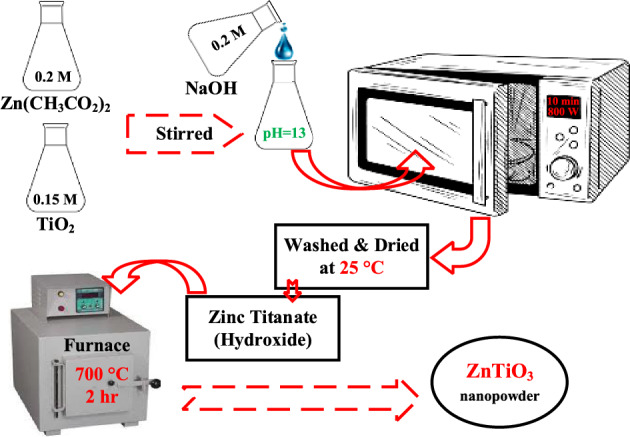
Preparing procedures of Zinc titanate nanostructure.

Figure 1 schematically illustrates the preparing procedures of ZnTiO_3_ nanostructures used as dopant in the interfacial polymer layer. In order to synthesize the ZnTiO_3_ nanostructures, at first, three beakers of Zn(CH_3_CO_2_)_2_, NaOH, and TiO_2_ solutions were prepared at 20^cc^ volume. The nanostructures of TiO_2_ have already been provided by dropwise adding 22^cc^ of TiCl_4_ (liquid phase) to 20^cc^ of NaOH solution on a magnetic stirrer and then irradiating with an 800 W microwave for 10 min. The obtained white mixture was rinsed by deionized water and dried at 25 °C. Next, the prepared solutions of TiO_2_ and NaOH were dropwise added to the Zn(CH_3_CO_2_)_2_ solution under an ultrasonic irradiation. Next, the produced solution was exposed by an 800 W microwave radiation for 10 min. Then, the result was rinsed by a centrifugation process and dried at 25 °C. At last, the resulted nano-powder was annealed at a temperature of 700 °C for 2 h.

### Washing process of Si wafer

In order to coat the interfacial polymer layer and make an MPS SD, the surface of the Si wafer needs to be cleaned from the pollutants as well as eliminate the SiO_2_ intrinsic layer on the surface. To this aim, different solutions have been used to wash the Si wafer in this work. First, it was rinsed by CH_3_COCH_3_ and CH_3_OH at a temperature of 55 °C for 5 minutes. Second, the Si wafer has been washed by a solution of H_2_O, NH_4_OH, and H_2_O_2_ at a temperature of 70 °C for 15 min. Finally, it was washed using a solution of H_2_O and HF acid at a temperature of 25 °C for 2 minutes.

### Fabrication of SDs

**Figure 2:**
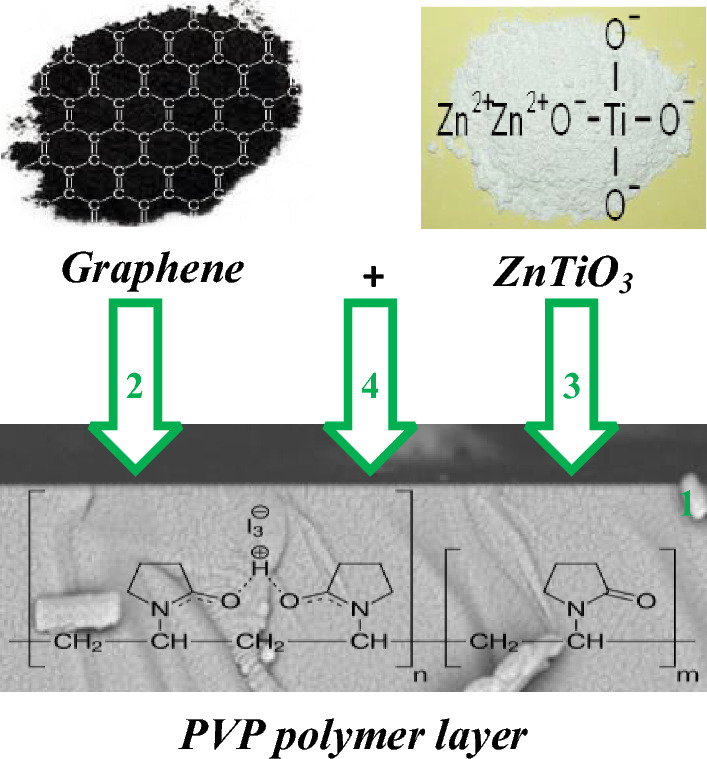
Preparation procedures of different SDs.

It is necessary to note that a 300 µm p-type Si wafer has been applied in this work. As an ohmic contact, an aluminum layer with a thickness of 100 nm was coated on the back side of silicon wafer at 10^–6^ Torr and then annealed at 500 °C. To make PVP:ZnTiO_3_ interfacial polymer layer, 10 mg of ZnTiO_3_ nanostructures was dispersed by ultrasonic technique after preparing a 5% solution of PVP with solvating 5 g of PVP nano-powders in 95^cc^ water. The preparing steps of different interfacial polymer layers are schematically presented in Fig. 2.

**Figure 3:**
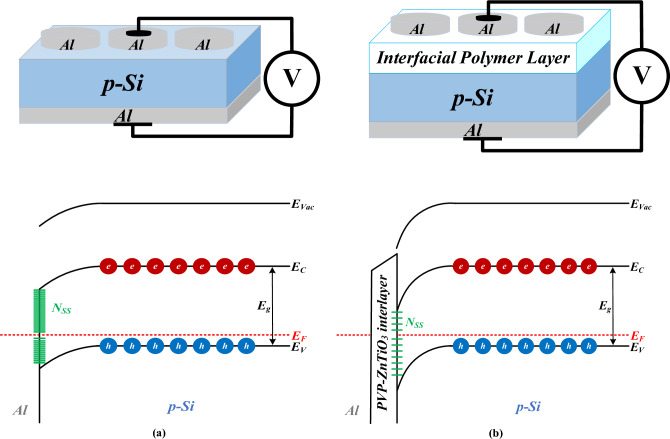
Schematic of the manufactured (**a**) MS and (**b**) MPS3 type-SDs and their corresponding energy-band diagrams.

Then, a soft layer of each prepared nanocomposites with a thickness of 100 nm has been deposited on the front side of the silicon wafer by using a spin coater system. At last, the 100 nm masks with 1.2 mm diameter were deposited on each prepared nanocomposite layers as the ohmic contacts. So, five SDs were manufactured by MS and MPS contacts without/with related interfacial nanocomposite layers. Figure 3 schematically shows these contacts with their corresponding energy-band diagrams.

There is several information on the grown and crystalline size, surface morphology, bandgap energy of the ZnTiO_3_ and HOMO and LUMO contour maps of PVP molecule can be found in our previous study^21^.

## ML algorithms

Machine learning, one of the substantial branches of artificial intelligence, is an interdisciplinary topic based on mathematics, statistics, computer science, and engineering, which optimized the computer programs' performance using data or former experiences. Several related research indicates that, the usage of machine learning is an effective method to get swiftly laws and trends from accessible data without needing the physical mechanism and also spending high experimental costs. Recent investigations demonstrated that machine learning has been utilized in various scientific and engineering problems such as biology, physics, chemistry, computer vision, medical care, industry, and even financial fields^22–32^. In this study, some machine learning techniques such as GPR, KRR, SVR, and ANN are used to model I–V diagram and predict some electrical and dielectric parameters of Schottky structures. Then the results obtained from the mentioned algorithms are compared with each other and with the experimental data.

In the following, we briefly explain the algorithms used in this research.

### GPR algorithm

GPR algorithm is a non-parametric, supervised machine learning technique that utilizes a probabilistic approach to interpreting data^33^. In GPR models, it is assumed that the outputs have a joint Gaussian distribution, providing a powerful tool for predicting outcomes^34^. So, the GPR problem can be formulated by estimating the probability distribution of the predicted variables given in the training data. It is able to define the joint probability distribution of the outputs as:

1 $$\left(\genfrac{}{}{0pt}{}{Y}{{Y}_{p}}\right)\sim \mathcal{N}(0,\updelta )$$

with $\mathcal{N}$ being the Gaussian distribution function with the mean value of **0** and the covariance matrix of δ. The mean value of the joint distribution will be assumed zero without loss of generality. It must be noted that the zero mean value can be deducted from the joint distribution to meet the assumption even if the output distribution is around some non-zero mean. The covariance matrix, δ, is given by;

2 $$\updelta =\left(\genfrac{}{}{0pt}{}{\begin{array}{cc}\mathrm{K}& {\mathrm{K}}_{*}\end{array}}{\begin{array}{cc}{\mathrm{K}}_{*}^{\mathrm{T}}& {\mathrm{K}}_{**}\end{array}}\right)$$

where K, K_∗_, and K_∗∗_ refer to the single covariance matrices which are the combinations of the training and test data sets as: K = K(X_train_; X_train_), K_∗_  = K(X_train_; X_test_), and K_∗∗_ = K(X_test_; X_test_). The covariance matrices, K, is described by a positive definite kernel function:

3 $$\mathrm{K}\left(\mathbf{x},{\mathbf{x}}^{\mathbf{^{\prime}}}\right)=-\lambda \mathrm{exp}(\frac{1}{2{\sigma }^{2}}{\Vert \mathbf{x}-{\mathbf{x}}^{\mathbf{^{\prime}}}\Vert }_{2}^{2})$$

where *σ* and *λ* denote hyperparameters of the GPR model with x and ${\mathrm{x}}^{\mathrm{^{\prime}}}$ being the input pairs depending on the suitable data sets. In fact, the distance or similarity between the input pairs are measured by the kernel function. The GPR aims to predict the new distribution of the test data based on the training data. Therefore, the Bayes’ rule is applied respectively to acquire the expected value and covariance of this new distribution as follows^33^:

4 $$E\left({\mathrm{Y}}_{\mathrm{p}}\left|\mathrm{Y}\right., {\mathrm{X}}_{\mathrm{train}},{\mathrm{X}}_{\mathrm{test}}\right)={\mathrm{K}}_{*}{\mathrm{K}}^{-1}\mathrm{Y}$$

5 $$\updelta \left({\mathrm{Y}}_{\mathrm{p}}\left|\mathrm{Y}\right., {\mathrm{X}}_{\mathrm{train}},{\mathrm{X}}_{\mathrm{test}}\right)={\mathrm{K}}_{**}-{\mathrm{K}}_{*}{\mathrm{K}}^{-1}{\mathrm{K}}_{*}$$

The predicted output distribution with the GPR is entirely defined by expressions (4) and (5). It is crucial that, unlike linear regression, GPR is not a parametric function. This non-parametric nature allows GPR to predict non-linear behaviors. Moreover, the GPR model not only indicates the expected value of the prediction but also provides an associated variance, giving confidence bounds on the model's predictions. Rather than relying solely on the training data, GP explicitly utilizes it to make predictions.

### KKR algorithm

Ridge regression is one of the elementary algorithms that are able to be kernelized. For this, it is necessary to find a linear function modeling the dependency of both continuous covariates {*x*_*i*_} and response variable {*y*_*i*_}. Minimizing the quadratic cost is the classical method to do that as^35^:

6 $$C\left({\varvec{w}}\right)=\frac{1}{2}\sum_{i}{({y}_{i}-{{\varvec{w}}}^{T}{{\varvec{x}}}_{i})}^{2}$$

The variable of *x*_*i*_ should be replaced by *ϕ*(*x*_*i*_) when working in the feature space. However, it leads to the risk of facing overfit, which avoiding it needs to regularize. An effective approach to regularization is to penalize the magnitude of the weights (**w**). Cross-validation or leave-one-out estimates are among the most commonly employed algorithms. Therefore, the total cost function which must be minimized is as follows^35^.

7 $$C=\frac{1}{2}\sum_{i}{({y}_{i}-{{\varvec{w}}}^{T}{{\varvec{x}}}_{i})}^{2}+\frac{1}{2}\lambda {\Vert {\varvec{w}}\Vert }^{2}$$

The following expression is acquired by solving derivatives and setting them equal to zero yields.

8 $$\sum_{i}({y}_{i}-{{\varvec{w}}}^{T}{{\varvec{x}}}_{i}){{\varvec{x}}}_{i}=\lambda {\varvec{w}} \Rightarrow {\varvec{w}}={\left(\lambda \mathbf{I}+ \sum_{i}{{\varvec{x}}}_{i}{{\varvec{x}}}_{i}^{T}\right)}^{-1}(\sum_{j}{y}_{j}{{\varvec{x}}}_{j})$$

It is clear that the regularization term results in numerically stabilizing the inverse by restricting the smallest eigenvalues from zero. When all data points replace by their feature vector, i.e. ${x}_{i}\to {\phi }_{i}=\phi ({{\varvec{x}}}_{i})$, the number of dimensions will be much larger than that of data points. It is able to use a clever technique to achieve the inverse in the most efficient way possible, either by reducing the dimensionality of the feature space or the number of data points. This technique is given by the following identity^36^:

9 $${({P}^{-1}+{B}^{T}{R}^{-1}B)}^{-1}{B}^{T}{R}^{-1}=P{B}^{T}{(BP{B}^{T}+R)}^{-1}$$

By defining $\phi ={\phi }_{ai}$ and ${\varvec{y}}={y}_{i}$, the solution is as follows:

10 $$w={(\lambda {\mathbf{I}}_{\mathbf{d}}+\phi {\phi }^{T})}^{-1}\phi {\varvec{y}}=\phi {({\phi }^{T}\phi +\lambda {\mathbf{I}}_{\mathbf{n}})}^{-1}{\varvec{y}}$$

This equation can be expressed in an alternate form as ${\varvec{w}}=\sum_{i}{\alpha }_{i}\phi ({{\varvec{x}}}_{i})$ by $\alpha ={({\phi }^{T}\phi +\lambda {\mathbf{I}}_{\mathbf{n}})}^{-1}{\varvec{y}}$. It must be demonstrated that it does not actually require access to the feature vectors, which could potentially be infinitely long. Practically, we require the predicted value for a new test case, **x**, which is determined with its projection on the solution, **w**,

11 $$y={{\varvec{w}}}^{T}\phi \left({\varvec{x}}\right)={\varvec{y}}{\left({\phi }^{T}\phi +\lambda {\mathbf{I}}_{\mathbf{n}}\right)}^{-1}{\phi }^{T}\phi (x)=y{\left(K+\lambda {\mathbf{I}}_{\mathbf{n}}\right)}^{-1}{\varvec{\kappa}}({\varvec{x}})$$

with $K\left(b{x}_{i},b{x}_{j}\right)={\phi }^{T}({x}_{i})\phi ({x}_{j})$ and ${\varvec{\kappa}}\left({\varvec{x}}\right)=K({{\varvec{x}}}_{i},{\varvec{x}})$^32^. So, it is only required access to the kernel K. There are various kernel functions such as linear, polynomial, sigmoid, and Radial Basis Function (RBF) which the last one has been selected in this work owing to the fewer factors and numerical complications of RBF^37^.

### ANN algorithm

A multilayer perceptron (MLP) employs a supervised-learning technique known as backpropagation to train the network, allowing it to learn from its mistakes and adjust its parameters accordingly. A MLP is composed of multiple layers of interconnected nodes, where each node (except for the input nodes) is a neuron with a nonlinear activation function^38^. These nodes are connected by a directed graph, forming a powerful network of neurons. In a MLP, neurons in two adjacent layers are connected via the weighted edges to form a pair. A MLP consists of at least three layers of neurons, including an input layer, one or more hidden layers, and an output layer. These layers are interconnected, allowing information to flow from the input layer to the output layer, helping the MLP to learn and make predictions^39^. The perceptron takes a linear combination of weighted real-valued inputs and passes it through a nonlinear activation function to generate an output, y, as^38^:

12 $$y=\varphi (\sum_{i=1}^{n}{w}_{i}{x}_{i}+b)$$

where b, x, w, and ϕ refer to the bias, the input vector, weights vector, and the activation function, respectively. The MLP algorithms commonly select the hyperbolic tangent, the logistic sigmoid function, and ReLU function as the activation functions^36^. The MLP algorithm adjusts the weights of the hidden layer in order to minimize the output error. By considering the difference between actual (O_n_(t)) and desired (T_n_) values, the error function could be written as^38^;

13 $$E\left({O}_{n}\left(t\right)\right)={T}_{n}-{O}_{n}\left(t\right)$$

In fact, minimizing the error function is the main propose of training procedure. By utilizing a learning parameter η (< 1), the convergence rate can be influenced and the step sizes at which weights are adapted can be reduced. The following rule can be used to update the ith weight connected to the jth output^38^:

14 $${w}_{ij}\left(t+1\right)-{w}_{ij}\left(t\right)=\eta E({O}_{j}\left(t\right))$$

Equations (1, 2, 3) illustrates an iterative weight adaptation process, in which a portion of the output error from the next iteration (t + 1) is added to the weight from the current iteration (t). MLPs are frequently employed for supervised-learning pattern recognition tasks. Interest in MLP backpropagation networks has been reignited due to the remarkable achievements of deep learning^40^. In this work, MLP was implemented with ten hidden layers and 100 neurons at each one. The input layer consists of applied voltage and different interfacial layers while the output layer includes the electric current.

### SVR algorithm

The Support Vector Method (SVM) is one of the machine-learning techniques proposed by Vapnik^41^. This method is a supervised learning model based on statistical learning that is used for analyzing existing data for classification and regression analysis. SVM training algorithm using given training data that have been labeled as belonging to one of two different classes, designs a model that determines which classification class each new data belongs to.

SVM maps data to a feature space of high dimension to classify them. Of course, sometimes data may not be linearly separable. Anyway, a hyperplane is used as a separator between the categories and passes through as many data points as feasible within a specified distance so-called the margin^42^. Consequently, the error in the prediction can be reduced and the non-linear relevance between input and target variables can be handled by the SVR algorithm using a kernel function.

Consider a training data set $T=\left\{\left({x}_{1},{y}_{1}\right), \dots , \left({x}_{N},{y}_{N}\right)\right\}$ including *N* ordered pairs of (*x*_*i*_*,y*_*i*_) for *i* = 1,2,…,*N*, where ${x}_{i}$ and ${y}_{i}$ imply the features and their corresponding values known as target values, respectively. For each feature (${x}_{i}$) a predicted value (${y}_{p}$) is considered to be fitted with $f\left(x\right)$ in the SVR algorithm. So, finding a smooth regression profile $f\left(x\right)$ with the minimum deviation ε value between the predicted and target values for all the data in the training set is the main proposal in implementing SVR. The estimation function of SVR algorithm, $f\left(x\right)$, could be given by^39^:

15 $$f\left(x\right)={w}^{T}\varphi \left(x\right)+b,$$

where w, $\varphi \left(x\right)$, and b are the weight vector, the feature function of input *x*, and a constant, respectively. In order to obtain the suitable regression function, it requires solving the convex optimization problem as follows:

16 $${\text{min }}\frac{1}{2}\left\| w \right\|^{2} s.t. \left\{ {\begin{array}{*{20}c} {y_{i} - w^{T} \varphi \left( {x_{i} } \right) - b \le \varepsilon ,} \\ {w^{T} \varphi \left( {x_{i} } \right) + b - y_{i} \le \varepsilon .} \\ \end{array} } \right.$$

**Figure 4:**
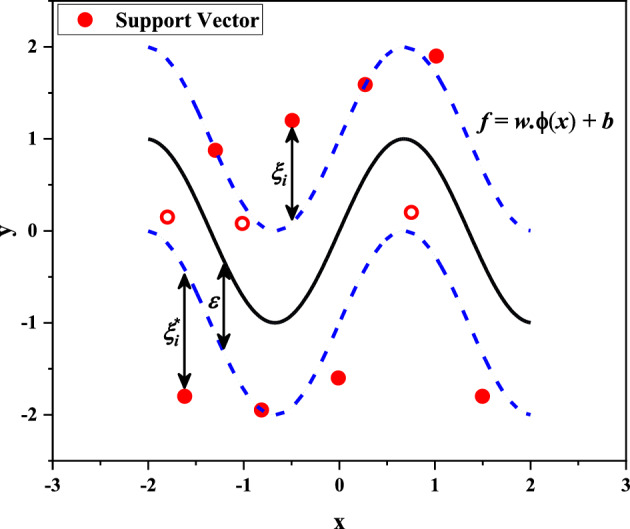
The structural parameters of SVR algorithm.

It should be mentioned that such function $f\left(x\right)$ satisfying these constraints for all points might not be found. The following positive and negative slack parameters ${\xi }_{i}$ and ${{\xi }_{i}}^{*}$ at every point can be presented to overcome to the infeasible constraints while the required conditions satisfy^39^:

17 $$\min \frac{1}{2}\left\| w \right\|^{2} + C\mathop \sum \limits_{i = 1}^{N} \left( {\xi_{i} + \xi_{i}^{*} } \right) s.t. \left\{ {\begin{array}{*{20}c} {y_{i} - w^{T} \varphi \left( {x_{i} } \right) - b \le \varepsilon + \xi_{i} ,} \\ {w^{T} \varphi \left( {x_{i} } \right) + b - y_{i} \le \varepsilon + \xi_{i}^{*} ,} \\ \end{array} } \right.$$

where $C$ is a predetermined penalty balancing the model complexity and the training set error and helping to prevent overfitting and ${\xi }_{i},{{\xi }_{i}}^{*}\ge 0$ for all *i*. The structural parameters of SVR algorithm are illustrated in Fig. 4.

This optimization problem can be solved by its converting to the dual problem at Karush–Kuhn–Tucker (KKT) condition^39^:

18 $$\begin{gathered} \max - \frac{1}{2}\mathop \sum \limits_{i,j = 1}^{N} \left( {\beta_{i} - \beta_{i}^{*} } \right)\left( {\beta_{j} - \beta_{j}^{*} } \right)\varphi \left( {x_{i} } \right)^{T} \varphi \left( {x_{j} } \right) - \varepsilon \mathop \sum \limits_{i = 1}^{N} \left( {\beta_{i} + \beta_{i}^{*} } \right) + \mathop \sum \limits_{i = 1}^{N} y_{i} \left( {\beta_{i} - \beta_{i}^{*} } \right) \hfill \\ s.t. \left\{ {\mathop \sum \limits_{i = 1}^{N} \left( {\beta_{i} - \beta_{i}^{*} } \right) = 0,} \right. \hfill \\ \end{gathered}$$

where ${\beta }_{i} ,{\beta }_{i}^{*}\in \left[0,C\right]$ are the Lagrange multipliers. Then, the SVR function could be expressed as follows after solving the dual problem:

19 $$f\left(x\right)=\sum_{i=1}^{N}\left({\beta }_{i}-{\beta }_{i}^{*}\right)k\left({x}_{i},{x}_{j}\right)+b,$$

where $k\left({x}_{i},{x}_{j}\right)={\varphi \left({x}_{i}\right)}^{T}\varphi \left({x}_{j}\right)$ refers to the kernel function allowing us to linearly solve the non-linear problems^40^. It must be noted that the RBF is also chosen in implementing the SVR algorithm^39,42^. So, it is given by:

20 $$k\left({x}_{i},{x}_{j}\right)=exp\left(-\gamma {\Vert {x}_{i}-{x}_{j}\Vert }^{2}\right)$$

where $\gamma$ denote the kernel width. The design of each ML models is carried out in three steps. The determination of hyper parameters forming the models is the first step. The analysis of training and predicting performance is the next step of the SVR model design. At last, the prediction data is obtained by the validated model.

All above algorithms have been used to compare their performance in predicting the electric current of the SDs with different interfacial layers.

### Error and accuracy functions

As mentioned, a detailed study on the prediction proficiency is necessary after completing the design of ML models. Also, the calculation of performance factors and then their analysis help us to understand the prediction accuracy. So, the examination accuracy of ML models used in this work is specified by the parameters of mean absolute error (MAE) and mean squared error (MSE) which are defined as follow^43^:

21 $$MAE=\frac{1}{N}\sum_{i=1}^{N}\left|{X}_{\mathrm{exp}(i)}-{X}_{pred(i)}\right|$$

22 $$MSE=\frac{1}{N}\sum_{i=1}^{N}{({X}_{\mathrm{exp}\left(i\right)}-{X}_{pred(i)})}^{2}$$

**Figure 5:**
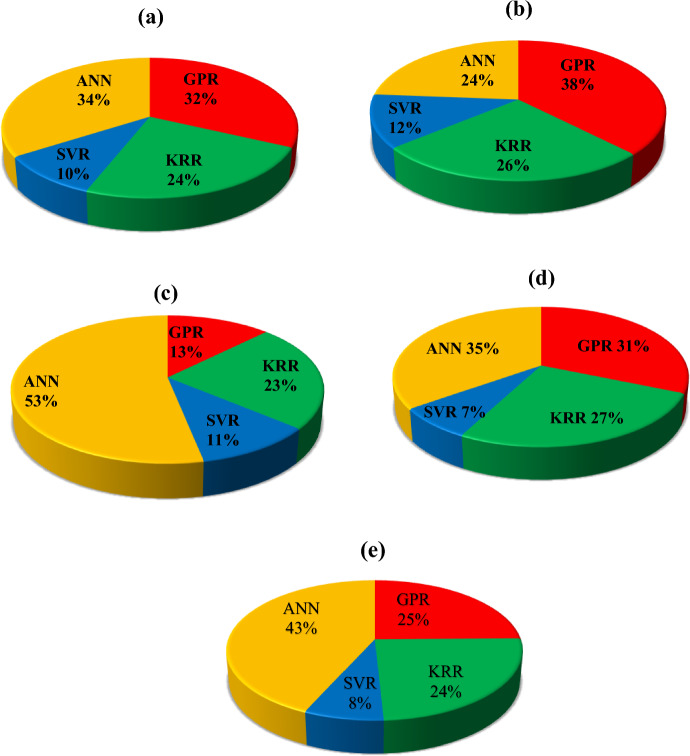
The MAE percentage (relative to one) of different algorithms in the prediction of current values for the (**a**) MS, (**b**) MPS1, (**c**) MPS2, (**d**) MPS3, and (**e**) MPS4 SDs.

Figure 5 show the MAE percentages of different algorithms applied for predicting the current value of the SD with and without interfacial layers. The values of MAE for each algorithm have been evaluated relative to 1. It is obvious that the MAE value of SVR model is lower than that of other models in the current prediction of all devices. Table 1 introduces the MSE value of each algorithm applied on the I–V characteristics of different MPS type-SDs to compare their accuracies in predicting the electric current with each other. The MSE value of the SVR model for all SDs is lower than that of the GPR, KRR, and ANN models. Although this value for the SVR algorithm is higher than that of the GPR and KRR ones at the MPS2 SD, the difference among them is small, and these values very close to each other.

**Table 1:** The MSE values of different algorithms in predicting the electric current of the prepared SDs.

SD	MSE			
	GPR	KRR	SVR	ANN
MS	0.0162	0.0099	0.0032	0.0107
MPS1	0.0172	0.0096	0.0026	0.0070
MPS2	0.0108	0.0206	0.0081	0.0200
MPS3	0.0146	0.0124	0.0027	0.0138
MPS4	0.0132	0.0127	0.0039	0.0117

**Figure 6:**
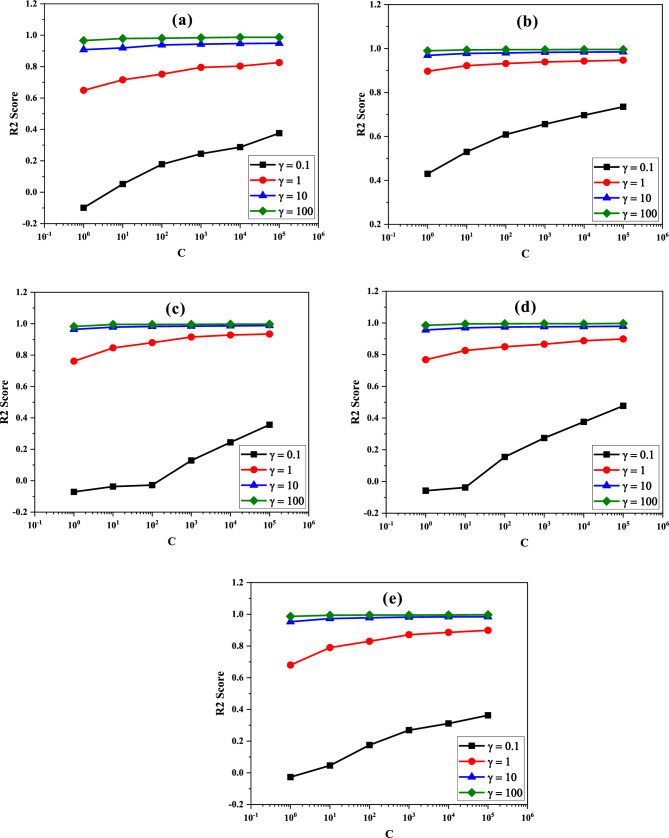
Variations of the R2 score vs the hyperparameters (C & γ) in the SVR model for the (**a**) MS, (**b**) MPS1, (**c**) MPS2, (**d**) MPS3, and (**e**) MPS4 SDs.

One of the other parameters that shows the accuracy of the used algorithm is the R2 score. Generally, the closer the value of the R2 score is to one, it means that the hyperparameters used in the algorithm are selected suitably and the more perfectly the model is trained, leading to predicting with less MSE. If the R2 score is equal to zero, the model would perform badly on an unseen dataset, originating from the bad selection of hyperparameters. The model is perfect provided that the value of the R2 score is equal to one. It is calculated as^44^:

23 $$R2\,\mathrm{Score}=1-\frac{\sum_{i=1}^{N}{({X}_{pred\left(i\right)}-{X}_{\mathrm{exp}\left(i\right)})}^{2}}{\sum_{i=1}^{N}{({\overline{X} }_{\mathrm{exp}(i)}-{X}_{\mathrm{exp}(i)})}^{2}}$$

where X_pred_ is the prediction value by the model, X_exp_ is the experimental value (actual value), and ${\overline{X} }_{exp}$ is the average value of the experimental data. In this work, the R2 score has only been determined for the SVR model due to its fewer MAE and MSE values among the used algorithms. Figure 6 shows the R2 score of the SVR model as a function of the hyperparameters used in this ML algorithm, i.e., C and γ. As seen, the value of the R2 score for all samples is becoming higher with increasing the values of C and γ in the SVR algorithm. The best value of these hyperparameter is C = 10^5^ and γ = 10^2^, resulting in the R2 score of 0.987 for MS, 0.995 for MPS1, 0.996 for MPS2, 0.997 for MPS3, and 0.998 for MPS4 SDs.

## Results and discussion

**Figure 7:**
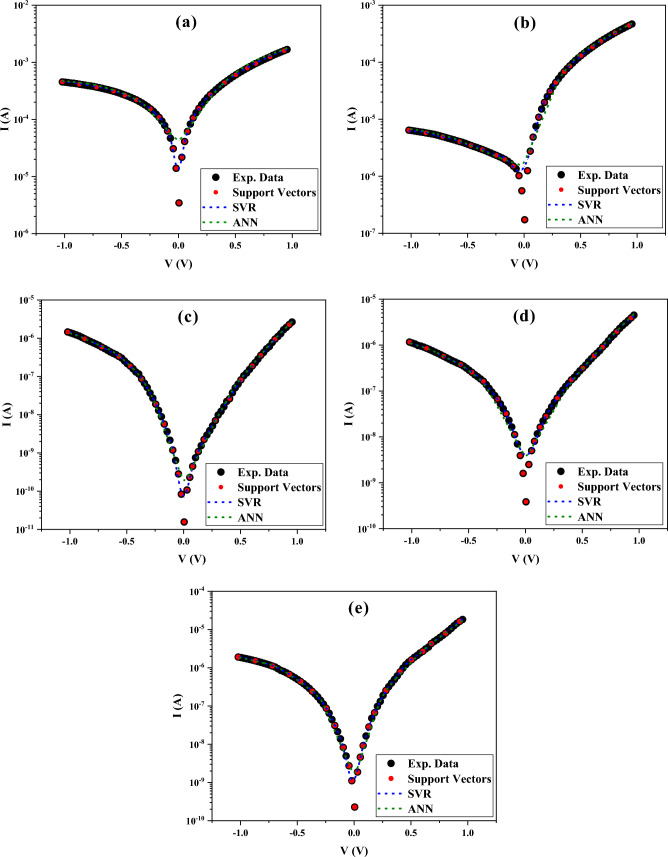
I–V characteristics of (**a**) MS, (**b**) MPS1, (**c**) MPS2, (**d**) MPS3, (**e**) MPS4 SDs and their analysis with the SVR and ANN models.

Figure 7 logarithmically shows the I–V characteristics of the prepared SDs with different interfacial layers at the voltage range of ± 1 V by using the experimental data, SVR and ANN models. It should be mentioned that the test size of the used algorithms was 80, which is selected so that predictions are made in the voltage range of ± 1 V. Moreover, the thermionic emission (TE) technique is chosen to determine the I–V characteristics of the prepared SDs because the ML method needs relatively high numbers of data to accurately predict the new data, in contrary to other techniques such as Norde, dV/dlnI, and H(I). It is worth mentioning that TE theory is based on the idea that the BH must be much greater than kT, meaning that only electrons with enough energy to surpass the potential barrier are taken into account when calculating the current density. Additionally, thermal equilibrium is assumed to be established at the plane that determines emission, and the presence of a net current flow does not disrupt this equilibrium^45^. As seen, the ability of the SVR model to predict the electric current of SDs at distinct biases is more reliable than other algorithms, especially the ANN model in confirming their MSE values (see Table 1).

**Figure 8:**
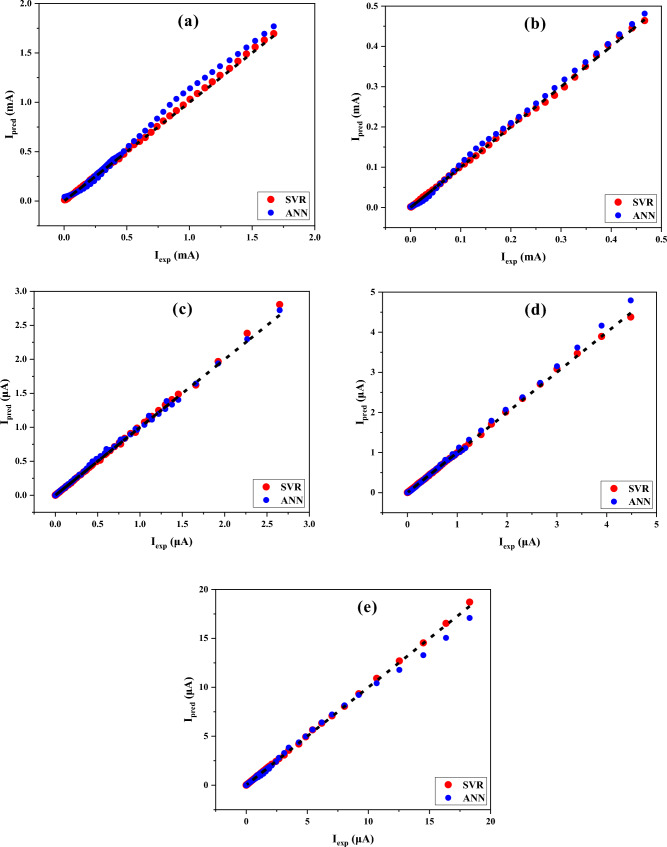
The predictive values of the SVR and ANN models for (**a**) MS, (b) MPS1, (**c**) MPS2, (**d**) MPS3, (**e**) MPS4 SDs.

In order to compare the prediction performance of the SVR and ANN models with each other, the actual and predictive values for each SD with different interfacial layers are simultaneously illustrated in Fig. 8. The experimental and output data are located on the x- and y-axis, respectively. To introduce a more detailed understanding of prediction accuracy, it is necessary to estimate the locations of data points. For this aim, the zero-error line has been plotted in Fig. 8 for a better examination of the data points’ location. The location of data points of the SVR and ANN models concerning the zero-error line is able to be another confirmation of the capability of the SVR model in accurately predicting the electrical current. And so, the electrical parameters of SDs with different interfacial layers is higher than the ANN model.

At the forward bias voltage and by considering a series resistance (R_s_) for V ≥ 3kT/q, the current–voltage relation in a SD could be expressed as^46^:

24 $$I={I}_{0}\left[\mathrm{exp}\left(\frac{q\left(V-I{R}_{s}\right)}{nkT}\right)-1\right],$$

where q is the electric charge (~ 1.60 × 10^–19^ C), k refers to the Boltzmann constant (~ 1.23 × 10^–23^ kg m^2^/K.s^2^), and T being the temperature (room temperature). The value of I_0_ is calculated by the linear part of ln(I)–V profile at V = 0 as^47^:

25 $${I}_{0}=A{A}^{*}{T}^{2}\mathrm{exp}\left(-\frac{q{\varphi }_{B0}}{kT}\right),$$

with A, A^*^, and ${\varphi }_{B0}$ being contact space, the Richardson constant, and BH at V = 0, respectively. Table 2 presents the values of I_0_ predicted by the ML algorithms for different prepared SDs in addition to their actual values. It is clear that the predicted value of I_0_ with the SVR model is the closest value to the actual one among other algorithms. Moreover, the values predicted by ANN algorithm are the farthest values from the actual ones for MPS1, MPS2, and MPS3 SDs. The GPR model has not predicted a suitable value for MS and MPS4 SDs compared to the actual value. It should be mentioned that the quantity of I_0_ for MS SD decreases with the interfacial layer usage, especially with graphene dopant. It implies the graphene nanoparticles doped in PVP polymer have a better influence in improving the electric features of MS SD.

**Table 2:** The actual and predicted values of I_0_ for different prepared SDs.

SD	I_0_ (A)				
	Actual	GPR	KRR	SVR	ANN
MS	6.2522 × 10^–5^	4.3619 × 10^–5^	6.1284 × 10^–5^	6.2469 × 10^–5^	4.4058 × 10^–5^
MPS1	5.1837 × 10^–6^	5.5595 × 10^–6^	5.5595 × 10^–6^	5.1586 × 10^–6^	2.2831 × 10^–6^
MPS2	2.5240 × 10^–10^	2.5494 × 10^–10^	3.3732 × 10^–10^	2.5195 × 10^–10^	1.5934 × 10^–10^
MPS3	5.9493 × 10^–9^	6.5095 × 10^–9^	5.9493 × 10^–9^	5.9493 × 10^–9^	3.5018 × 10^–9^
MPS4	8.1114 × 10^–9^	1.0840 × 10^–8^	7.6389 × 10^–9^	8.0933 × 10^–9^	5.3831 × 10^–9^

The primary purpose of using a nanocomposite interfacial layer is to control and engineer the potential BH of the MS SD. So, it is one of the important electrical parameters of SDs that should be calculated at zero-bias voltage as^48^:

26 $${\varphi }_{B0}=\frac{kT}{q}\mathrm{ln}\left(\frac{A{A}^{*}{T}^{2}}{{I}_{0}}\right).$$

The BHs of various prepared SDs have been obtained and given in Table 3. Additionally, ML algorithms have been utilized to model the electric current used for calculating the BH of different SDs. In this case, the prediction of almost all algorithms is in good agreement with the actual value, while the SVR model has predicted an exact value corresponding to the actual one. From a physical point of view, the BH of MS contact has been raised when an interfacial polymer layer is put at the M/S interface. The highest increment is realized depending on the MPS2 SD, whose nanocomposite interfacial layer is doped by graphene nanoparticles.

**Table 3:** The actual and predicted values of φ_B_ for different prepared SDs.

SD	φ_B_(eV)				
	Actual	GPR	KRR	SVR	ANN
MS	0.5957	0.6049	0.5961	0.5954	0.6047
MPS1	0.6601	0.6583	0.6583	0.6608	0.6813
MPS2	0.9169	0.9167	0.9095	0.9170	0.9289
MPS3	0.8352	0.8328	0.8352	0.8352	0.8489
MPS4	0.8272	0.8197	0.8288	0.8282	0.8378

The ideality factor is one of the other investigated electrical parameters of diodes in this research, which determines how the behavior of the diode follows the ideality diode. Its quantity (n) is unity at the ideal case although it generally deviated from the ideal conditions because of the presence of native or deposited interfacial layers such as polymer, insulator, and Ferro-electric materials at the M/S interface. Furthermore, the density of surface states formed at the interface of the interfacial layer and semiconductor, the thickness of the depletion layer (W_d_ = (2ε_s_ε_o_V_i_/qN_a_)^0.5^) related to the concentration of donor or acceptor atoms doped into the pure semiconductor, the thickness and dielectric value of the interfacial layer are the other effective parameters in determining the ideality factor as [n = 1 + d_i_/$\varepsilon$_i_ ($\varepsilon$_i_/W_d_ + qN_ss_)]. With the use of the slope of ln(I)–V profile, the quantity of n is given by^21,49^:

27 $$n=\frac{q}{kT}\left(\frac{dV}{d\left(lnI\right)}\right).$$

It must be noted that other processes such as generation/recombination of electron–hole pairs, electrons tunneling via the potential barrier by the interface states, and barrier lowering owing to image-force influence on the ideality factor value and current-transport/conduction mechanisms in the MPS devices^1,50^. Table 4 shows the value of n calculated for the prepared SDs with and without different interfacial layers between the metal and semiconductor. Its reduction from 7.69 for MS to 3.31 for MPS4 implies that the existence of the interfacial layer leads to getting close the behavior of SD to the ideal case. Again, the ML algorithms have been applied to predict the n value for the considered SDs. It is obvious that the SVR model was able to calculate the ideality factor of SDs with high accuracy. Although other algorithms have suitable predictions in some cases, their deviation from the actual values is more than the SVR model. Among the ML algorithms considered in this work, the worst performance is related to the ANN model due to the highest deviation of prediction values from the corresponding actual ones.

**Table 4:** The actual and predicted values of n for different prepared SDs.

SD	n				
	Actual	GPR	KRR	SVR	ANN
MS	7.6987	6.0766	7.6077	7.7051	6.5615
MPS1	5.2869	5.5369	5.5289	5.2869	4.0384
MPS2	3.3174	3.1993	3.4912	3.3202	2.9212
MPS3	4.4991	4.6284	4.4835	4.4944	4.0724
MPS4	3.3060	3.5951	3.2531	3.3031	3.0217

Series resistance (R_s_) is an electric parameter of diodes that affects the performance of SDs, especially the rectifying ratio (RR). It originated from several different sources such as the probe wires, the ohmic and rectifier contacts made on the back and front sides of the semiconductor bulk, the resistance of semiconductor, inhomogeneities of the dopant donor or acceptor atoms at the semiconductor, remaining impurities from the cleaning process between the contacts^1,51–53^. In spite of the fact that the series resistance is able to be neglected at the inversion and depletion regions, it will be more effective in the accumulation region^1,2^. In this work, the ohmic law is used to calculate the value of R_s_ and R_sh_ as follows^52^:

28 $${R}_{i}=\frac{d{V}_{i}}{d{I}_{i}}.$$

The quantity of R_s_ obtained for the prepared SDs with/without interfacial layer at the voltage of + 3 V is presented in Table 5. The ML algorithms are also applied to predict the R_s_ value of the considered SDs. As can be observed, the predictions of all algorithms are in good agreement with the actual values, however, the SVR model is able to predict the R_s_ quantity corresponding to the actual values with the minimum differences. Moreover, the prediction deviation of the KRR algorithm from the actual values is smaller than the GPR model and the ANN algorithm shows the most deviation from the actual value of R_s_ for different SDs.

**Table 5:** The actual and predicted values of R_s_ for different prepared SDs.

SD	R_s_(kΩ)				
	Actual	GPR	KRR	SVR	ANN
MS	0.3002	0.3025	0.3021	0.3008	0.3138
MPS1	0.4331	0.4425	0.4303	0.4313	0.4112
MPS2	1.0447	1.0396	1.0415	1.0438	1.0547
MPS3	1.4389	1.4389	1.4390	1.4388	1.3264
MPS4	0.6423	0.6410	0.6409	0.6419	0.7189

There is a shunt resistance (R_sh_) at the reverse bias voltage originating from the probe wire-ground patches, imperfections at the contact area, and leakage current across the interfacial layer^53^. Besides, the applied voltage (V_a_) will be shared across the MPS structure possessing R_s_ and N_ss_ as V_a_ = V_i_ + V_Rs_ + V_d_ + V_ss_, resulting in the deviation of lnI–V profile from the linearity form at sufficient large forward bias voltages^54^. Also, the value of R_sh_ for the various prepared SDs with/without interfacial layer is calculated at the reverse bias voltage of -3 V and room temperature represented in Table 6. Using the ML algorithms, the quantity of R_sh_ has been predicted with different accuracies to compare with the actual values.

**Table 6:** The actual and predicted values of R_sh_ for different prepared SDs.

SD	R_sh_(kΩ)				
	Actual	GPR	KRR	SVR	ANN
MS	3.8922	3.8844	3.8768	3.8910	3.7781
MPS1	173.82	173.58	173.59	173.78	160.51
MPS2	305.19	304.19	304.12	305.03	314.10
MPS3	495.28	494.93	494.61	495.22	489.62
MPS4	345.09	343.86	343.89	344.97	335.27

The SVR predictions of the R_sh_ values for the considered SDs are closest to the actual ones while the ANN model shows the maximum deviation from the actual values among the applied ML algorithms. The GPR and KRR models are able to predict the R_sh_ value with the minimum deviation from the actual ones. From the physical point of view, the interfacial layer can drastically increase the shunt resistance from 3.89 kΩ for the MS type-SD to 495 kΩ for the MP3 type-SD, resulting in the leakage reduction of oxide/current ways at the interfacial layer and hence enhancement the performance of SDs.

The rectifying rate of the SD is determined by the quantity of RR which can be defined with the ratio of the electric current at the forward bias voltage (+ 3 V) to the reverse bias voltage (− 3 V) as RR = I_F_/I_R_^55^. Table 7 demonstrates the RR value of different prepared SDs in this work at room temperature. It is observable that the RR of the MS structure is significantly enhanced by inserting an interfacial layer between the metal and semiconductor, especially for MPS4-type SD whose interfacial layer has been doped by Gr and ZnTiO_3_ nanostructures. Since the ML algorithms have predicted the electric current of the considered SDs at the bias voltage range of ± 3 V, the predicted electric currents at the forward and reverse bias voltages are able to be used for calculating the RR values of different SDs. The RR values obtained based on the prediction values of the SVR model have the most agreement with the actual values. Besides, the lowest deviation in the calculation of RR quantities is related to the GPR and KRR predictions while the most deviation corresponds to the ANN prediction. Some experimental and theoretical studies on the structural, electrical, and optical features of these structures were also reported in the literature in the last years^56–60^.

**Table 7:** The actual and predicted values of RR for different prepared SDs.

SD	RR				
	Actual	GPR	KRR	SVR	ANN
MS	12.963	12.840	12.833	12.936	12.039
MPS1	401.34	392.81	403.42	402.92	390.35
MPS2	292.12	292.59	291.99	292.23	297.80
MPS3	344.19	343.94	343.72	344.18	369.12
MPS4	537.32	536.42	536.56	537.42	466.29

One of the reasons of deviating the SD from the ideal behavior is the inhomogeneity distribution of metal/oxide dopants in the semiconductor along with the interface states. The following method could be used to understand how the importance of this flaw; the dependence of N_ss_ on the voltage at the equilibrium condition is described as^1,2^:

29 $${N}_{ss}\left(V\right)=\frac{1}{q}\left[\frac{{\varepsilon }_{i}}{\delta }\left(n\left(V\right)-1\right)-\frac{{\varepsilon }_{s}}{{W}_{D}}\right],$$

with W_D_ and δ being the depletion layer width and the thickness of the interfacial layer (~ 100 nm). Moreover, *ε*_*i*_ and *ε*_*s*_ refer to the interlayer/semiconductor permittivity, respectively. It must be noted that the energy difference of the *N*_*ss*_ level and valance band for a p-type semiconductor is written as^1–3^:

30 $${E}_{ss}-{E}_{v}=q\left({\varphi }_{e}-V\right).$$

By considering the relation among R_s_, *φ*_*e*_, and n(V) could be derived as^3^:

31 $${\varphi }_{e}-{\varphi }_{B0}=\left(1-\frac{1}{n\left(V\right)}\right)V.$$

Using the Eqs. (13, 14, 15), the *N*_*ss*_ values can be calculated for different prepared SDs represented in Table 8. It can be seen that inserting the interfacial layer with and without dopants between the metal and semiconductor leads to decreasing the density of interface states, especially for MPS2 where the PVP polymer layer is doped by the graphene nanostructures. Also, these graphene nanostructures significantly reduce the *N*_*ss*_ of the MPS4 whose interfacial layer has been doped by the Gr-ZnTiO_3_ nanostructures compared with the MPS3 with the ZnTiO_3_ nanostructures doped in the PVP polymer layer. It is owing to the fact that the interfacial layer is led to a passivation at the semiconductor surface^52^. The energy-band diagrams representing the reduction of *N*_*ss*_ in the MS and MPS3 SDs schematically show in Fig. 3. It should be mentioned that the creation of BH and native or deposited polymer layer at the M/S interface, their homogeneity distribution, and surface-states (*N*_*ss*_) lead to the conduction mechanisms (CMs) in the SDs^3–5^. Furthermore, the ML algorithms are used to predict the value of *N*_*ss*_ for different SDs. The prediction values with the SVR model are the same as the actual values whereas the prediction of other algorithms is also in good agreement with the actual values. Therefore, almost all ML algorithms considered in this study are able to predict the density of interface states with high reliability.

**Table 8:** The actual and predicted values of N_ss_ for different prepared SDs.

SD	N_ss_ × 10^13^ (eV^1^.cm^-2^)				
	Actual	GPR	KRR	SVR	ANN
MS	6.8645	6.8999	6.8752	6.8634	6.8200
MPS1	4.7603	4.7777	4.7603	4.7593	4.7745
MPS2	0.1535	0.1534	0.1533	0.1535	0.1550
MPS3	1.4529	1.4528	1.4527	1.4529	1.4549
MPS4	0.4255	0.4256	0.4255	0.4255	0.4302

## Conclusions

In this research, five Schottky Diodes (SDs) with the structures of Al/p-Si (MS), Al/PVP/p-Si (MPS1), Al/PVP:Gr/p-Si (MPS2), Al/PVP:ZnTiO3/p-Si (MPS3), and Al/PVP:Gr-ZnTiO3/p-Si (MPS4) have been fabricated to investigate the effect of interfacial polymer layers with/without dopants on the basic electric parameters of the MS type SD. Then, the I–V features of the SDs have been studied by the TE method to calculate and analyze their principal electric parameters, such as I_0_, ${\varphi }_{B0}$, n, R_s_, R_sh_, RR, and N_ss_. All the above-mentioned parameters have been predicted by using some ML algorithms such as GPR, KRR, SVR, and ANN and compared each other. The obtained results show that the quantity of I_0_ or leakage current and N_ss_ for MS type SD decreases, and ${\varphi }_{B0}$ increases with the interfacial layer usage, especially with graphene dopant. On the other hand, the BH of MS contact raises when an interfacial polymer layer is put at the M/S interface. The highest increase of BH occurred for the MPS2 structure due to the nanocomposite interfacial layer doping with graphene nanoparticles. Hence, the graphene nanoparticles doped in the PVP polymer have a better influence for the improvement of the electrical specifications of MS type SD. The reduction of the ideality factor (n) from 7.69 for MS SD to 3.31 for MPS4 structure depicts that the behavior of SD becomes closer to the ideal case due to the existence of the interfacial layer. Also, the RR of the MS structure is significantly enhanced by inserting an interfacial layer between the metal and semiconductor, especially for MPS4 SD whose interfacial layer has been doped by Gr and ZnTiO_3_ nanostructures. The interface states density decreases for MPS2, MPS4, and MPS3 structures due to the existence of the PVP polymer layer between the metal and semiconductor doped by the Gr, Gr-ZnTiO_3_, and ZnTiO_3_ nanostructures, respectively. Comparing the actual and predicted values of I_0_ by the ML algorithms for different SDs demonstrates that the values predicted by the ANN algorithm are the farthest values from the actual ones for MPS1, MPS2, and MPS3 SDs. This also has occurred for data predicted by the GPR model for MS and MPS4 SDs. On the contrary, for all structures, the data predicted by the SVR is closer to the real data.

The BH prediction values by almost all algorithms are in good agreement with the actual values, while the SVR model has predicted closer values to the corresponding actual ones. The prediction performance of all used algorithms for ideality factor n, R_s_, and R_sh_ is almost suitable. Their deviation of prediction values from the actual ones, especially in the ANN model is more than the SVR model. The acquired RR values for different SDs are feasible with respect to the predicted electric currents at the forward and reverse biases because the electric current of the considered SDs has been predicted by the ML algorithms at the voltage range of ± 1 V. The predicted RR values by the SVR model have the most agreement with the actual values. Besides, the lowest deviation in the calculation of RR quantities is related to the GPR and KRR predictions while the most deviation corresponds to the ANN prediction. The deviation of N_ss_ prediction values from actual ones is low enough for all used algorithms, especially SVR. In conclusion, almost all ML algorithms considered in this study are able to predict the density of interface states with high reliability. Considering the SDs electric current predictions due to the MSE values of ML algorithms, the prediction ability of the SVR model is more reliable than the other algorithms, especially the ANN model. Moreover, the hyperparameters used in GPR, KRR, and SVR algorithms are fewer than the ANN model. So, not only are these algorithms easier to use, but algorithm optimization is easier due to having fewer hyperparameters. As a results, this research can give researchers ideas to conduct more studies to identify other more suitable algorithms as well as improve and optimize them as much as possible.

## Data Availability

The datasets used and/or analyzed during the current study are available from the corresponding author upon reasonable request.

## References

[1] Sze SM, Ng KK (2006). LEDs and lasers. Phys. Semicond. Devices.

[2] Nicollian, E. H., Brews, J. R. *MOS (metal oxide semiconductor) Physics and Technology*. 920 (John Wiley & Sons, 2002).

[3] Card HC, Rhoderick EH (1971). Studies of tunnel MOS diodes I. Interface effects in silicon Schottky diodes. J. Phys. D Appl. Phys..

[4] Tung RT (2014). The physics and chemistry of the Schottky barrier height. Appl. Phys. Rev..

[5] Al-Ahmadi NA (2020). Metal oxide semiconductor-based Schottky diodes: A review of recent advances. Mater. Res. Express.

[6] Altındal Ş, Barkhordari A, Azizian-Kalandaragh Y, Çevrimli BS, Mashayekhi HR (2022). Dielectric properties and negative-capacitance/dielectric in Au/n-Si structures with PVC and (PVC: Sm2O3) interlayer. Mater. Sci. Semicond. Process..

[7] Al-Dharob MH, Lapa HE, Kökce A, Özdemir AF, Aldemir DA, Altındal Ş (2018). The investigation of current-conduction mechanisms (CCMs) in Au/(0.07 Zn-PVA)/n-4H-SiC (MPS) Schottky diodes (SDs) by using (IVT) measurements. Mater. Sci. Semicond. Process..

[8] Altındal Yerişkin S, Balbaşı M, Orak İ (2017). The effects of (graphene doped-PVA) interlayer on the determinative electrical parameters of the Au/n-Si (MS) structures at room temperature. J. Mater. Sci. Mater. Electron..

[9] Çiçek O, Altındal Ş, Azizian-Kalandaragh Y (2020). A highly sensitive temperature sensor based on Au/graphene-PVP/n-Si type Schottky diodes and the possible conduction mechanisms in the wide range temperatures. IEEE Sens. J..

[10] Reddy VR, Prasad CV (2018). Surface chemical states, electrical and carrier transport properties of Au/ZrO2/n-GaN MIS junction with a high-k ZrO2 as an insulating layer. Mater. Sci. Eng., B.

[11] Ersöz G, Yücedağ İ, Azizian-Kalandaragh Y, Orak I, Altındal Ş (2016). Investigation of electrical characteristics in Al/CdS-PVA/p-Si (MPS) structures using impedance spectroscopy method. IEEE Trans. Electron Devices.

[12] Azizian-Kalandaragh Y (2010). Dielectric properties of CdS-PVA nanocomposites prepared by ultrasound-assisted method. Optoelectron. Adv. Mater. Rapid Commun..

[13] Houssein EH, Abohashima Z, Elhoseny M, Mohamed WM (2022). Machine learning in the quantum realm: The state-of-the-art, challenges, and future vision. Expert Syst. Appl..

[14] Torun Y, Doğan H (2021). Modeling of Schottky diode characteristic by machine learning techniques based on experimental data with wide temperature range. Superlattices Microstruct..

[15] Ali HA, El-Zaidia EF, Mohamed RA (2020). Experimental investigation and modeling of electrical properties for phenol red thin film deposited on silicon using back propagation artificial neural network. Chin. J. Phys..

[16] Güzel T, Çolak AB (2021). Artificial intelligence approach on predicting current values of polymer interface Schottky diode based on temperature and voltage: An experimental study. Superlattices Microstruct..

[17] Çolak AB, Güzel T, Yıldız O, Özer M (2021). An experimental study on determination of the shottky diode current-voltage characteristic depending on temperature with artificial neural network. Phys. B.

[18] Kim HT, Nahm S, Byun JD, Kim Y (1999). Low-fired (Zn, Mg) TiO_3_ microwave dielectrics. J. Am. Ceram. Soc..

[19] Gui Y, Li S, Xu J, Li C (2008). Study on TiO_2_-doped ZnO thick film gas sensors enhanced by UV light at room temperature. Microelectron. J..

[20] Durmus Z, Durmus A, Kavas H (2015). Synthesis and characterization of structural and magnetic properties of graphene/hard ferrite nanocomposites as microwave-absorbing material. J. Mater. Sci..

[21] Barkhordari A, Mashayekhi HR, Amiri P, Altındal Ş, Azizian-Kalandaragh Y (2023). Role of graphene nanoparticles on the electrophysical processes in PVP and PVP: ZnTiO_3_ polymer layers at Schottky diode (SD). Semicond. Sci. Technol..

[22] Crampon K, Giorkallos A, Deldossi M, Baud S, Steffenel LA (2022). Machine-learning methods for ligand–protein molecular docking. Drug Discov. Today.

[23] Chan CH, Sun M, Huang B (2022). Application of machine learning for advanced material prediction and design. EcoMat..

[24] Xu P, Chen H, Li M, Lu W (2022). New opportunity: Machine learning for polymer materials design and discovery. Adv. Theory Simul..

[25] Tao Q, Xu P, Li M, Lu W (2021). Machine learning for perovskite materials design and discovery. npj Comput. Mater..

[26] Liu X, Xu P, Zhao J, Lu W, Li M, Wang G (2022). Material machine learning for alloys: Applications, challenges and perspectives. J. Alloy. Compd..

[27] Sabry F, Eltaras T, Labda W, Alzoubi K, Malluhi Q (2022). Machine learning for healthcare wearable devices: The big picture. J. Healthc. Eng..

[28] Mueller B, Kinoshita T, Peebles A, Graber MA, Lee S (2022). Artificial intelligence and machine learning in emergency medicine: A narrative review. Acute Med. Surg..

[29] Ivanciuc O (2007). Applications of support vector machines in chemistry. Rev. Comput. Chem..

[30] Doğan H, Duman S, Torun Y, Akkoyun S, Doğan S, Atici U (2022). Neural network estimations of annealed and non-annealed Schottky diode characteristics at wide temperatures range. Mater. Sci. Semicond. Process..

[31] Güzel T, Çolak AB (2022). Investigation of the usability of machine learning algorithms in determining the specific electrical parameters of Schottky diodes. Mater. Today Commun..

[32] Ahmed S, Alshater MM, El Ammari A, Hammami H (2022). Artificial intelligence and machine learning in finance: A bibliometric review. Res. Int. Bus. Financ..

[33] Rasmussen CE, Williams CK (2006). Gaussian Processes for Machine Learning.

[34] Murphy KP (1991). Machine Learning.

[35] Mohri M, Rostamizadeh A, Talwalkar A (2018). Foundations of Machine Learning.

[36] Rupp M, Tkatchenko A, Müller KR, Von Lilienfeld OA (2012). Fast and accurate modeling of molecular atomization energies with machine learning. Phys. Rev. Lett..

[37] Zhang R, Wang W (2011). Facilitating the applications of support vector machine by using a new kernel. Expert Syst. Appl..

[38] Ahmadloo E, Azizi S (2016). Prediction of thermal conductivity of various nanofluids using artificial neural network. Int. Commun. Heat Mass Transfer.

[39] Awad M, Khanna R (2015). Efficient Learning Machines: Theories, Concepts, and Applications for Engineers and System Designers.

[40] Ali A, Abdulrahman A, Garg S, Maqsood K, Murshid G (2019). Application of artificial neural networks (ANN) for vapor-liquid-solid equilibrium prediction for CH4-CO2 binary mixture. Greenh. Gases Sci. Technol..

[41] Vapnik, V. N. *The Nature of Statistical Learning* (1998).

[42] Hsu, C. W., Chang, C. C., Lin, & C. J. *A Practical Guide to Support Vector Classification* 1396–1400 (2003).

[43] Çolak AB, Yıldız O, Bayrak M, Tezekici BS (2020). Experimental study for predicting the specific heat of water based Cu-Al_2_O_3_ hybrid nanofluid using artificial neural network and proposing new correlation. Int. J. Energy Res..

[44] Öcal S, Gökçek M, Çolak AB, Korkanç M (2021). A comprehensive and comparative experimental analysis on thermal conductivity of TiO_2_-CaCO_3_/Water hybrid nanofluid: Proposing new correlation and artificial neural network optimization. Heat Transf. Res..

[45] Potje-Kamloth K (2002). Chemical gas sensors based on organic semiconductor polypyrrole. Crit. Rev. Anal. Chem..

[46] Durmus Z, Durmus A, Kavas H (2015). Synthesis and characterization of structural and magnetic properties of graphene/hard ferrite nanocomposites as microwave-absorbing material. J. Mater. Sci..

[47] Barkhordari A, Altındal Ş, Pirgholi-Givi G, Mashayekhi H, Özçelik S, Azizian-Kalandaragh Y (2022). The influence of PVC and (PVC: SnS) interfacial polymer layers on the electric and dielectric properties of Au/n-Si structure. Silicon.

[48] Altındal Ş, Sevgili Ö, Azizian-Kalandaragh Y (2019). A comparison of electrical parameters of Au/n-Si and Au/(CoSO_4_–PVP)/n-Si structures (SBDs) to determine the effect of (CoSO_4_–PVP) organic interlayer at room temperature. J. Mater. Sci.: Mater. Electron..

[49] Vargas O, Caballero Á, Morales J (2014). Enhanced electrochemical performance of maghemite/graphene nanosheets composite as electrode in half and full Li–ion cells. Electrochim. Acta.

[50] Altındal Ş, Barkhordari A, Özçelik S, Pirgholi-Givi G, Mashayekhi HR, Azizian-Kalandaragh Y (2021). A comparison of electrical characteristics of Au/n-Si (MS) structures with PVC and (PVC: Sm2O3) polymer interlayer. Phys. Scr..

[51] Ashiri R, Nemati A, Ghamsari MS, Sanjabi S, Aalipour M (2011). A modified method for barium titanate nanoparticles synthesis. Mater. Res. Bull..

[52] Barkhordari A, Özçelik S, Altındal Ş, Pirgholi-Givi G, Mashayekhi H, Azizian-Kalandaragh Y (2021). The effect of PVP: BaTiO_3_ interlayer on the conduction mechanism and electrical properties at MPS structures. Phys. Scr..

[53] Ansaree J, Upadhyay S (2015). Thermal analysis of formation of nano-crystalline BaTiO_3_ using Ba(NO_3_)_2_ and TiO2. Process. Appl. Ceram..

[54] Yu P, Cui B, Shi Q (2008). Preparation and characterization of BaTiO_3_ powders and ceramics by sol-gel process using oleic acid as surfactant. Mater. Sci. Eng., A.

[55] Altındal Ş, Barkhordari A, Pirgholi-Givi G, Ulusoy M, Mashayekhi H, Özçelik S, Azizian-Kalandaragh Y (2021). Comparison of the electrical and impedance properties of Au/(ZnOMn: PVP)/n-Si (MPS) type Schottky-diodes (SDs) before and after gamma-irradiation. Phys. Scr..

[56] Rahman N, Husain M, Yang J, Sajjad M, Murtaza G, Ul Haq M, Habib A, Rauf A, Karim A, Nisar M, Yaqoob M (2021). First principle study of structural, electronic, optical and mechanical properties of cubic fluoro-perovskites:(CdXF3, X= Y, Bi). Eur. Phys. J. Plus.

[57] Husain M, Rahman N, Khan R, Zulfiqar S, Khattak SA, Khan SN, Sohail M, Iqbal A, Reshak AH, Khan A (2022). Structural, electronic, elastic, and magnetic properties of NaQF3 (Q= ag, Pb, Rh, and Ru) flouroperovskites: A first-principle outcomes. Int. J. Energy Res..

[58] Husain M, Rahman N, Khan R, Sohail M, Khan AA, Elansary HO, El-Abedin TK, Mahmoud EA, Abdelmohsen SA, Khan A (2022). Exploring the exemplary structural, electronic, optical, and elastic nature of inorganic ternary cubic XBaF3 (X= Al and Tl) employing the accurate TB-mBJ approach. Semicond. Sci. Technol..

[59] Saddique J, Husain M, Rahman N, Khan R, Iqbal A, Sohail M, Khattak SA, Khan SN, Khan AA, Reshak AH, Khan A (2022). Modeling structural, elastic, electronic and optical properties of ternary cubic barium based fluoroperovskites MBaF3 (M= Ga and In) compounds based on DFT. Mater. Sci. Semicond. Process..

[60] Husain M, Rahman N, Albalawi H, Ezzine S, Amami M, Zaman T, Rehman AU, Sohail M, Khan R, Khan AA, Khan A (2022). Examining computationally the structural, elastic, optical, and electronic properties of CaQCl 3 (Q= Li and K) chloroperovskites using DFT framework. RSC Adv..

